# Evaluation of a Multiparametric Immunofluorescence Assay for Standardization of Neuromyelitis Optica Serology

**DOI:** 10.1371/journal.pone.0038896

**Published:** 2012-06-12

**Authors:** Letizia Granieri, Fabiana Marnetto, Paola Valentino, Jessica Frau, Agata Katia Patanella, Petra Nytrova, Patrizia Sola, Marco Capobianco, Sven Jarius, Antonio Bertolotto

**Affiliations:** 1 Clinical Neurobiology Unit, Regional Referring Multiple Sclerosis Centre (CReSM), University Hospital San Luigi Gonzaga, Orbassano, Turin, Italy; 2 Neuroscience Institute Cavalieri Ottolenghi (NICO), University of Turin, Orbassano, Turin, Italy; 3 Multiple Sclerosis Centre, Department of Cardiovascular and Neurological Science, University of Cagliari, Cagliari, Italy; 4 Multiple Sclerosis Centre, Department of Neuroscience, Gemelli Hospital, Rome, Italy; 5 Department of Neurology and Center of Clinical Neuroscience, General University Hospital in Prague and First Faculty of Medicine, Charles University in Prague, Prague, Czech Republic; 6 Multiple Sclerosis Centre, S. Agostino Estense Hospital, Modena, Italy; 7 Division of Molecular Neuroimmunology, Department of Neurology, University of Heidelberg, Heidelberg, Germany; Innsbruck Medical University, Austria

## Abstract

**Background:**

Neuromyelitis optica (NMO) is a severely disabling autoimmune disorder of the central nervous system, which predominantly affects the optic nerves and spinal cord. In a majority of cases, NMO is associated with antibodies to aquaporin-4 (AQP4) (termed NMO-IgG).

**Aims:**

In this study, we evaluated a new multiparametric indirect immunofluorescence (IIF) assay for NMO serology.

**Methods:**

Sera from 20 patients with NMO, 41 patients with multiple sclerosis (MS), 30 healthy subjects, and a commercial anti-AQP4 IgG antibody were tested in a commercial composite immunofluorescence assay (“Neurology Mosaic 17”; Euroimmun, Germany), consisting of five different diagnostic substrates (HEK cells transfected with AQP4, non-transfected HEK cells, primate cerebellum, cerebrum, and optic nerve tissue sections).

**Results:**

We identified AQP4 specific and non-specific fluorescence staining patterns and established positivity criteria. Based on these criteria, this kit yielded a high sensitivity (95%) and specificity (100%) for NMO and had a significant positive and negative likelihood ratio (LR+ = ∞, LR− = 0.05). Moreover, a 100% inter- and intra-laboratory reproducibility was found.

**Conclusions:**

The biochip mosaic assay tested in this study is a powerful tool for NMO serology, fast to perform, highly sensitive and specific for NMO, reproducible, and suitable for inter-laboratory standardization as required for multi-centre clinical trials.

## Introduction

Neuromyelitis optica (NMO) is a severely disabling autoimmune disorder of the central nervous system, which mainly affects the optic nerves and spinal cord [1], [2]. In the majority of cases, NMO is associated with autoantibodies to the water channel aquaporin-4 (AQP4) (termed NMO-IgG) [3], [4]. Anti-AQP4 antibodies have also been found in patients with isolated longitudinally extensive transverse myelitis and in patients with isolated optic neuritis, conditions which are considered limited or inaugural forms of NMO [5]–[7]. In addition, anti-AQP4 antibodies have been found in a subset of patients with connective tissue disorders (CTD) such as lupus erythematosus (SLE), Sjogren's syndrome and co-existing NMO spectrum disorders (NMOSD) [8]–[10].

Since the discovery of anti-AQP4 antibodies, several assays for the detection of NMO-IgG have been developed [11]. However, most of these assays are available only at few specialized laboratories. Moreover, most of them lack independent standardization and validation, and no generally accepted gold standard assay exists.

The present study aimed to evaluate a new commercially available multiparametric indirect immunofluorescence (IIF) assay in distinguishing NMO from MS patients. This assay consists of an array of five different diagnostic substrates including HEK cells transfected with AQP4, non-transfected HEK cells, and three monkey tissue sections (cerebellum, cerebrum, and optic nerve). The assay was evaluated through the following steps: 1. Characterization of distinct immunofluorescence staining patterns. 2. Correlation between staining patterns and the patients' clinical diagnoses. 3. Evaluation of the diagnostic sensitivity, specificity, and clinical utility (as assessed by calculation of likelihood ratios) of each pattern. 4. Analysis of the assay's inter- and intra-laboratory reproducibility.

Our results show that this IIF assay has high sensitivity and specificity and represents a powerful tool for NMO serology, permitting the identification of different AQP4 specific and non-specific patterns. Moreover this assay is fast to perform, highly reproducible and suitable for inter-laboratory standardization.

## Materials and Methods

### Ethics Statement

This study was approved by the Ethical Committee of the San Luigi University Hospital (approval n. 1704). An informed written consent was obtained from each individual.

### Patients and Healthy Controls

Patients and controls were recruited from five MS centres at the following university hospitals: S. Luigi Gonzaga (Orbassano, Italy), Policlinico Gemelli (Rome, Italy), Binaghi (Cagliari, Italy), Modena (Italy), and Charles (Prague, Czech Republic).

Demographic and clinical characteristics of patients and controls are shown in Table 1. All samples were processed in a blinded fashion.

**Table 1:** Demographic and clinical characteristics of subjects.

		NMO (n = 20)	MS (n = 41)	HC (n = 30)
***Demography***				
Male/Female		2 (10%)/18 (90%)	16 (39%)/25 (61%)	14 (47%)/16 (53%)
Median age at blood withdrawal, years		45 (19–72)	32 (10–69)	32 (22–58)
Median age at onset, years		31(13–62)*	28 (5–54)*	
***Clinical features***				
Optic neuritis		20/20 (100%)	20/41 (49%)	
	*Monophasic*	5/20 (25%)	16/20 (80%)	
	*Recurrent*	15/20 (75%)	4/20 (20%)	
Transverse Myelitis		20/20 (100%)	19/41 (46%)	
	*Monophasic*	5/20 (25%)	14/19 (74%)	
	*Recurrent*	15/20 (75%)	5/19 (26%)	
***Imaging and CSF***				
Initial MRI brain, does not meet MS criteria**		20/20 (100%)	6/41 (15%)	
MRI spinal cord lesion ≥3 segments		20/20 (100%)	0/41 (0%)	
CSF positive for OB***		4/18 (22%)	34/37 (92%)	

NMO: neuromyelitis optica, MS: multiple sclerosis, HC: healthy controls, MRI: magnetic resonance imaging, CSF: cerebrospinal fluid, OB: oligoclonal bands.

* One NMO patient and one MS patient are pediatric.

** According to Wingerchuk 2006, where MRI Paty criteria for MS were included.

*** CSF data were not available for 2 NMO patients and for 4 MS patients.

#### Identification of NMO patients

**Figure 1:**
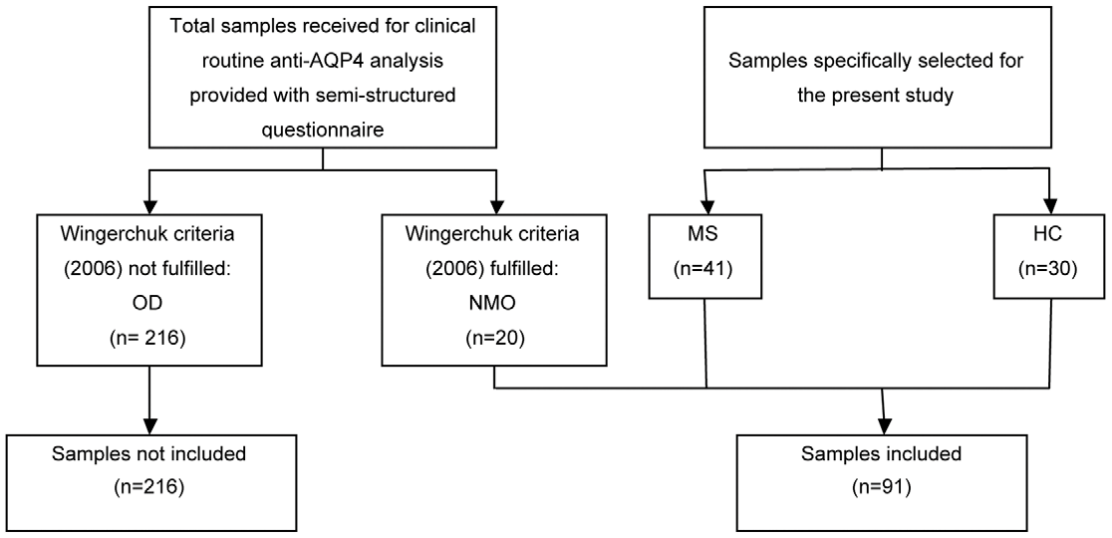
Recruitment of NMO patients, MS patients and HC. OD: Other diseases, NMO: neuromyelitis optica, MS: multiple sclerosis, HC: healthy controls.

NMO patients were selected from a total of 236 serum samples which our laboratory had received for diagnostic purpose in 2009 and 2010. Clinical data were provided by the senders using a semi-structured questionnaire containing the 2006 Wingerchuk criteria (i.e. the minor criterion of NMO-IgG seropositivity was not considered to avoid selection bias towards NMO-IgG positive cases) [2]. Based on the data reported in the questionnaires, 20/236 cases met the clinical and radiological criteria for NMO. These cases were classified as “clinically and radiologically defined NMO” (N = 20) and included in the present study (Figure 1). Sixteen of these 20 patients were treatment-free at the time of blood withdrawal (12 treatment-naïve, 1 previously treated with immunosuppressive drugs, and 3 previously treated with immunosuppressive and immunomodulant drugs); the remaining 4 NMO patients were under immunosuppressive (2×azathioprine) or immunomodulatory therapy (1×interferon-beta, 1×glatiramer acetate) at the time of blood withdrawal. Conversely, samples from patients who did not meet these criteria or from patients from whom no sufficient data was available to evaluate whether the criteria were met were excluded (N = 216).

#### Selection of MS patients and healthy controls

As controls, 41 patients with definite relapsing remitting multiple sclerosis (RRMS) according to the revised McDonald's criteria [12], naïve to any immunomodulatory therapy, and 30 healthy volunteers were enrolled. These samples were not selected from the 236 sera described above, but they were specially selected for the present study.

### BioChip Mosaic™ Indirect Immunofluorescence Assay (IIF)

Samples were tested for NMO-IgG using a multiparametric commercial IIF assay (“Neurology Mosaic 17”, Euroimmun, Luebeck, Germany); the 5 kits used for the present study were purchased by S. Luigi Gonzaga University Hospital. This assay is provided as a ready-to-use kit consisting of microscopy slides with five reactions fields, each containing an array of five different biological substrates (i.e. HEK cells transfected with AQP4-Ab, non transfected HEK cells, and primate cerebellum, cerebrum and optic nerve cryosections), positive and negative control samples, and a pre-diluted goat anti-human IgG secondary antibody conjugated to fluorescein isothiocyanate (FITC). The substrates are applied to coated cover glasses by the manufacturer, which are then automatically cut to millimetre-sized fragments (termed biochips) and transferred to the reaction fields. This approach allows simultaneous testing of patient samples on several substrates. Briefly, 25 µl of a 1∶60 diluted serum samples were applied to each reaction field according to the manufacturer's protocol. After 30 min of incubation at room temperature, the slides were washed with PBS-Tween (0,002%) for 5 minutes. Then 20 µl of fluorescein-labelled anti-human IgG were applied to each reaction field and incubated with the BioChip slides for 30 minutes. After an additional 5 min wash with PBS-Tween (0,002%), a glass coverslip was applied to each slide. The mounting medium contained an antifading agent and 4,6-diamidino-2-phenylindole (DAPI) for nuclear staining (ProLong Gold with DAPI, Invitrogen Ltd., Renfrew, UK). Sections were analysed under a DMIRE2 Leica fluorescence microscope (Leica, Milan, Italy) with a 40× oil immersion lens. Pictures were acquired with a digital camera model DC250 Leica, using the acquisition software Qfluor550 Leica.

**Figure 2:**
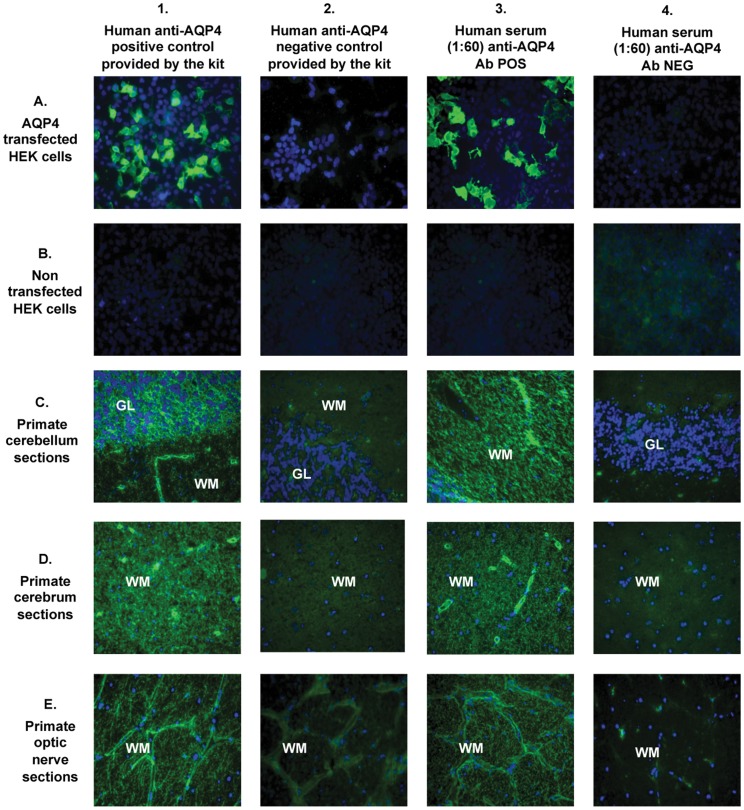
Fluorescence staining patterns as observed with positive and negative controls provided by the manufacturer (column 1 and 2, respectively), an anti-AQP4 antibody positive human serum sample (column 3), and an anti-AQP4 antibody negative human control sample (column 4). The biochip mosaic consists of 5 substrates: HEK cells transfected with full length recombinant human AQP4 (row A), non-transfected HEK cells (row B) and cryosections of primate cerebellum (row C), cerebrum (row D) and optic nerve (row E). Bound IgG was visualized using secondary antibodies labeled with FITC (green). Cell nuclei, stained with DAPI, are shown in blue. Magnification 40×. GL: granular layer; WM: white matter; DAPI: 4,6-diamidino-2-phenylindole; FITC: fluorescein isothiocyanate.

Positive and negative human control sera provided by the manufacturer were tested in each working session; in addition, a goat polyclonal anti-AQP4 IgG (H19, sc-9887, Santa Cruz Biotechnology Inc., Santa Cruz, CA) was tested as a supplemental positive control in a 1∶ 500 dilution. All samples and controls were tested in a blinded fashion by two different operators (LG and FM).

### Evaluation of the Assay

#### Characterization of fluorescence staining patterns

Based on anatomic and morphological characteristics, a number of typical staining patterns were obtained by incubating the various substrates with i.) a commercial goat anti-human AQP4 IgG antibody, ii.) anti-AQP4 IgG antibody positive control sera (included in the IIF kit), iii.) 91 serum samples from patients with NMO and controls.

**Figure 3:**
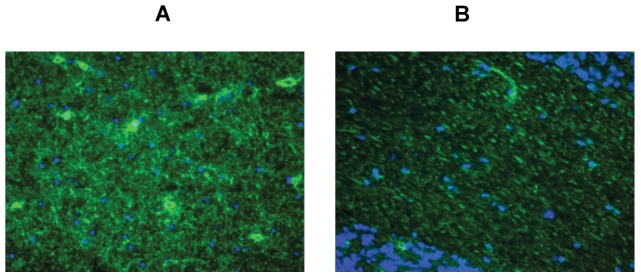
“Typical” (A) and “atypical” (B) white matter fluorescence staining as observed with anti-AQP4 positive NMO (A) and anti-AQP4 negative MS (B) human serum samples, respectively. Bound IgG was visualized using secondary antibodies labeled with FITC (green). Cell nuclei, stained with DAPI, are shown in blue. Magnification 40×. NMO: neuromyelitis optica; MS: multiple sclerosis; DAPI: 4,6-diamidino-2-phenylindole; FITC: Fluorescein isothiocyanate.

#### Comparison between fluorescence patterns and patients' clinical diagnosis

The different fluorescence staining patterns were associated to the clinical status of the subjects (NMO, MS, or healthy).

#### Reproducibility analysis

To evaluate the assay's intra-laboratory reproducibility, positive and negative control samples included in the kit were tested in 20 independent runs. Moreover, each serum sample was tested in our laboratory twice by two different operators in a blinded fashion (LG and FM). Furthermore, the inter-laboratory reproducibility was evaluated by blinded testing of 9 serum samples in our laboratory and at the Institute for Experimental Immunology, Euroimmun, Luebeck, Germany.

**Figure 4:**
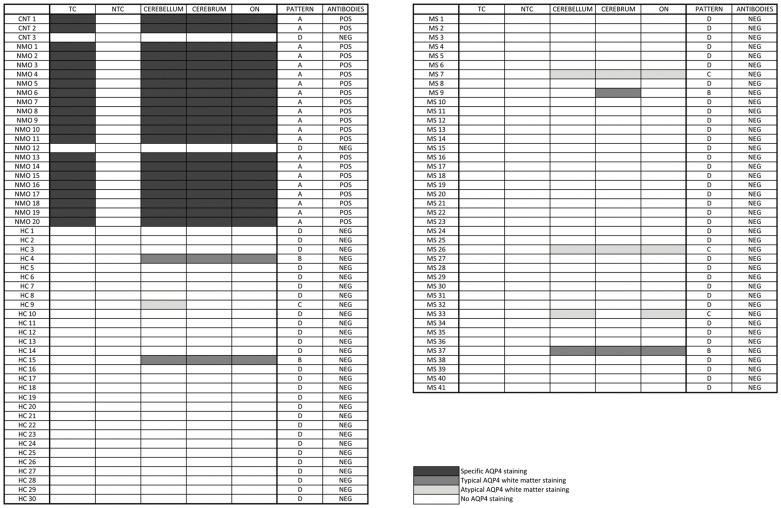
Differential distribution of IIF staining patterns in five diagnostic substrates following incubation with serum samples from patients with NMO or controls. The corresponding staining patterns (A, B, C, D; typical and atypical white matter staining), as defined in the results section, are indicated, together with the final evaluation of positivity or negativity for anti-AQP4 antibodies, and the healthy volunteers' clinical status. NMO: neuromyelitis optica, MS: multiple sclerosis, HC: healthy controls, CNT 1: commercial goat polyclonal anti-human AQP4 IgG (H19, Santa Cruz Biotechnology), CNT 2: human anti-AQP4 positive serum provided by the manufacturer, CNT 3: human anti-AQP4 negative serum provided by the manufacturer, TC = transfected cells, NTC = non-transfected cells, ON = optic nerve.

#### Statistical analyses

Sensitivity and specificity for each pattern were calculated using the clinical and MRI diagnosis of NMO as reference standard. Clinical utility of each substrate was then evaluated by comparing likelihood ratios (LR) [13] for positive (sensitivity divided by 1- specificity) and negative (1- sensitivity divided by specificity) test results. LRs of >10 for a positive test result or <0.1 for a negative test result are expected to yield a conclusive change in the post-test odds of disease presence. The inter-rater agreement kappa test was used to evaluate the agreement between paired combinations of results and centers for the case of positive and negative classification (ordinal outcomes): K coefficient is an index of agreement, ranging from 0 (no agreement beyond chance) to/1 (perfect agreement), calculated as a measure of agreement corrected for chance [14], [15]. All statistical analyses were realized using the GraphPad Prism® Program Version 4.0 (GraphPad Software Inc., San Diego, CA, USA).

## Results

### Characterization of staining patterns

#### Definition of anti-AQP4 specific and non-specific fluorescence staining

The anti-AQP4 positive human serum (included in the kit) and a commercial goat polyclonal anti-AQP4 IgG antibody were used as positive controls to define the anti-AQP4 specific staining for each substrate (Figure 2: 1A, 1B, 1C, 1D, 1E). In the cell membrane of the AQP4 transfected HEK cells the antibodies formed a flat, smooth, or fine granular fluorescence, whereas no staining was found with the non-transfected HEK cells (Figure 2: 1A, 1B). Characteristic NMO-IgG staining of the pia mater (when present in the tissue section) and microvasculature in the gray and white substance was observed (Figure 2: 1C, 1D, 1E). In addition, the extracellular spaces in the granular layer of primate cerebellum were strongly stained resulting in a mesh wire-like pattern (Figure 2: 1C). Finally, the white matter of primate cerebrum, cerebellum, and optic nerve showed staining of a very dense and irregular network of fine filamentous structures, which was defined as “typical AQP4 white matter staining” (Figure 2: 1C, 1D, 1E; Figure 3A).

An atypical white matter staining was observed with some sera from MS patients and HC, which was characterized by a regular network of filamentous structures, predominantly orientated in parallel with the axons (Figure 3B).

#### Definition of four distinct staining patterns

By testing 91 serum samples, four distinct staining patterns were identified. Their complete spectrum is represented in Figure 4.

*Pattern A* was characterized by the presence of staining of the cell membrane of the AQP4-transfected HEK cells in the absence of staining of the membrane of the non-transfected HEK cells, mesh wire like staining in the granular layer, pia mater (when present) staining and the “typical AQP4 white matter staining” as described above in all primate tissue sections. (Figure 2: 3A, 3B, 3C, 3D, 3E).
*Pattern B* was characterized by the presence of the “typical AQP4 white matter staining” as described above in one or more primate tissue sections in the absence of any other staining (Figure 2: 4A, 4B, 3C, 3D, 3E).
*Pattern C* was characterized by the presence of the “atypical white matter staining” as described above detectable on one or more primate tissues in the absence of any other staining (Figure 3B, Figure 2: 4A, 4B).
*Pattern D* was defined by the complete absence of staining in all five substrates (Figure 2: 2A, 2B, 2C, 2D, 2E; 4A, 4B, 4C, 4D, 4E).

### Association between fluorescence patterns and patients' clinical diagnosis

We associated the four different patterns with the clinical status (NMO, MS, or healthy) of each subject. Pattern A was found in 19/20 (95%) serum samples from patients diagnosed with NMO according to clinical and MRI findings, and it was not observed with any MS sample nor with any healthy control sample (HC). Pattern B and C were present in a subset of control patients. Pattern D was associated with NMO in only one single case but was frequently observed with the MS and HC controls. See Table 2 for details.

**Table 2:** Association between fluorescence patterns and clinical status of all the analyzed samples (2 commercial anti-AQP4 positive controls, 20 NMO samples, 41 MS samples, 30 healthy subjects).

	POSITIVE CONTROLS	NMO	MS	HC
**PATTERN A**	100% (2/2)	95% (19/20)	0% (0/41)	0% (0/30)
**PATTERN B**	0% (0/2)	0% (0/20)	5% (2/41)	7% (2/30)
**PATTERN C**	0% (0/2)	0% (0/20)	7% (3/41)	3% (1/30)
**PATTERN D**	0% (0/2)	5% (1/20)	88% (36/41)	90% (27/30)

NMO: neuromyelitis optica, MS: multiple sclerosis, HC: healthy controls.

### Sensitivity, specificity and likelihood ratios (LR)

The clinical and MRI diagnostic criteria for NMO [2] were considered as the “gold standard” for sensitivity, specificity, and LR analyses for each pattern. Pattern A showed 95% sensitivity and 100% specificity for NMO. Accordingly, pattern A had a very high positive LR (LR+, ∞; should be >10 to be clinically useful) and a very low negative likelihood ratio (LR−, 0.05; should be <1). To note, pattern A did not include the microvasculature staining, which has been so far considered a typical NMO-IgG feature: in fact, it was observed in 9/20 (45%) NMO patients (the patient negative for pattern A was also negative for microvasculature staining), and in 4/71 control sera (1 MS and 3 HC), thus showing 45% sensitivity and 95% specificity for NMO. Patterns B and C showed no sensitivity for NMO, while Pattern D showed 5% sensitivity.

Sensitivity, specificity, and LR for NMO were calculated also by considering the “typical AQP4 white matter staining” in each single substrate of biochip mosaic. All substrates showed 95% sensitivity for NMO and a LR− of 0.05, but the AQP4-transfected HEK cells had a higher specificity (100%) and LR+ (∞) than the other substrates (specificity, 94–96%; LR+, 15.83–23.75). Results are summarized in Table 3.

**Table 3:** Diagnostic sensitivity, specificity and likelihood ratios (LR+, LR−) for NMO calculated for each single substrate of the BioChip, obtained by testing 91 serum samples on the “Neurology Mosaic 17”.

	SENSITIVITY FOR NMO	SPECIFICITY FOR NMO	LR+	LR−
**AQP4 TRANSFECTED CELLS**	95%	100%	∞	0.05
**PRIMATE CEREBELLUM (Typical AQP4 white matter staining)**	95%	96%	23.75	0.05
**PRIMATE CEREBRUM (Typical AQP4 white matter staining)**	95%	94%	15.83	0.05
**PRIMATE OPTIC NERVE (Typical AQP4 white matter staining)**	95%	96%	23.75	0.05

NMO: neuromyelitis optica, LR: likelihood ratio.

### Reproducibility

#### Intra-laboratory reproducibility

To evaluate the assay's intra-laboratory inter-run and inter-rater reproducibility, positive and negative control samples (provided by the manufacturer) were tested by two blinded operators (LG, FM) in 20 independent runs. In addition, all 91 serum samples were tested and evaluated in a blinded fashion in our laboratory by the two operators (LG, FM) in different working sessions. A 100% inter-rater (K = 1) and a 100% inter-run agreement (K = 1) was found.

#### Inter-laboratory reproducibility

Nine serum samples (4 from NMO patients and 5 from HC) provided by the Multiple Sclerosis Centre at the Charles University Hospital (Prague, Czech Republic) were tested in parallel in our laboratory (CReSM, Orbassano, Turin) and at the Institute of Experimental Immunology, affiliated to Euroimmun, in Luebeck, Germany, to evaluate the assay's inter-laboratory variability. Raters at both laboratories were blinded to the donors' clinical status. A 100% concordance between laboratories was found (K = 1).

## Discussion

NMO serology has become an important aspect in the diagnostic workup of patients with NMO and has been included in the revised diagnostic criteria for this condition [2]. More recently, anti-AQP4 antibodies have been found also in a subset of patients with isolated transverse myelitis [5], patients with isolated optic neuritis [16], [6], [7], and patients with NMOSD and co-existing CTD [8]–[10], leading to an increase in the number of clinical conditions that require testing for anti-AQP4 antibodies.

Testing for anti-AQP4 antibodies is important not only also from a diagnostic but also from a therapeutic point of view, since treatment options differ considerably between NMO and MS. Immunomodulatory drugs (i.e. interferon beta, natalizumab and fingolimod) are believed to be preferential in MS, while their use could be detrimental in NMO. In particular, interferon beta was shown to trigger severe disease exacerbation in patients with NMOSD [17], [18]. Similarly, a failure of natalizumab to control disease activity in anti-AQP4 antibody positive NMOSD patients was reported [19], [20]. Very recently, extensive brain lesions were reported in an anti-AQP4 antibody positive patient following treatment with fingolimod (FTY720) [21]. In contrast, immunosuppressive drugs such as azathioprine, rituximab, or mycophenolate, which are not among the first line treatments for MS, have been shown to be effective in NMO [22]–[26].

Over the last couple of years, several assays have been developed for the detection of anti-AQP4 antibodies [11] including indirect immunohistochemistry (IHC) on mouse or monkey brain sections [3], [27], [28], cell based assays (CBA) [28]–[30], a radioimmunoprecipitation assay [31], fluoroimmunoprecipitation assays [30], [32], an enzyme linked immunosorbent assay [33], FACS based assays [34], [35], and western blot analysis [36]. However, the sensitivity and specificity of these assays differed markedly (sensitivity ranges from 33% to 91%, specificity from 85% to 100%) [11]. Moreover, some methods have yielded discordant results when applied at different laboratories, and incongruous results were found with identical samples tested in different assays in some studies [32]–[34], suggesting a possible lack of standardization and validation of the various in-house assays currently available. Accordingly, none of these methods has been so far generally accepted as a “gold standard” or reference method.

However, highly standardized and reproducible assays for the detection of anti-AQP4 antibodies are crucial for large multi-centre studies aiming to better define the epidemiological, clinical, and pathological features of patients with NMO and their response to treatment with respect to the patients anti-AQP4 antibody serostatus, as previously pointed out by Fazio and colleagues [37]. Moreover, only if standardized assays are applied, results are comparable between studies.

The aim of the present study was to perform a systematic evaluation of a new multiparametric indirect immunofluorescence assay (“Neurology Mosaic 17”, Euroimmun, Luebek, Germany). This assay potentially meets some of the requirements for future multicentre trials. First, as a commercial assay it is not restricted to a few specialized laboratories as many of the in house-assays used in previous studies and thus available for independent evaluation. Second, as a commercial assay is produced in a standardized way, potentially reducing the variability frequently associated with in-house assays. Third, cells and tissues are produced by the manufacturer at large scale and stored in liquid nitrogen until array assembly; this potentially allows eliminating assay variability due to changes over time in transfection rates or in tissue quality. Fourth, the assay is relatively simple to perform and less time-consuming and labour-intensive if compared to immunoprecipitation or FACS analysis, which require rather sophisticated techniques such as cell culture and cell transfection to be available at the performing laboratory. Moreover, the assay can potentially be used to obtain semi-quantitative results on anti-AQP4 antibodies titres. Monitoring AQP4-Ab titre dynamics over time could be important, since recent evidence indicates that anti-AQP4 titers might correlate with disease activity in NMO [11], [29], [38], though we could not test the latter point due to a lack of suitable follow-up samples. Finally, the use of biochips allows assessing several substrates (transfected cells, brain tissue sections) in a single session, eliminating the need for multiple incubations and reducing the total serum volume required to 2 µl. These features render the assay a possible candidate for future trials in NMO.

To evaluate the assay's diagnostic performance, we tested a series of 91 serum samples (20 NMO patients, 41 MS patients and 30 HC), positive and negative controls provided by the manufacturer, and a commercial antibody to human AQP4 to define AQP4-Ab specific and non-specific staining patterns on the various substrates included in the biochip. This led us to the identification of 4 different fluorescence patterns (termed A, B, C, D). Of particular note, we identified two distinct patterns of white matter staining, which may be helpful in the interpretation of fluorescence patterns on primate brain sections as they help avoiding false positive results. By correlating the various fluorescence patterns observed with the clinical status of the analysed subjects, we established pattern A as the only staining pattern that is highly specific for AQP4. This pattern was found with both positive controls and with 19/20 sera from patients with NMO, corresponding to a sensitivity of 95% for NMO. Sensitivity, specificity and LRs were evaluated also for each substrate separately, i.e. for the transfected cells and the three brain tissue sections. All substrates showed the same sensitivity (95%) for NMO, but different specificity values. While the transfected cells had a 100% specificity, the three monkey tissues showed lower specificity rates due to the presence of AQP4-like white matter positive staining in some MS patients and HC (Table 3). Given the fact that all established characteristics of AQP4 antibody-specific staining were missing and the CBA was negative, the presence of white matter staining in these rare MS and HC patients is likely to indicate the presence of serum antibodies directed against so far unknown antigens other than AQP4.

Sensitivity, specificity and LR values obtained by using either the whole biochip mosaic or the transfected cells substrate alone were the same. Formally, the cell-based assay would thus be sufficient for the detection of antibodies against AQP4. However, this multiparametric assay allows to test the same sample simultaneously on different substrates and by two different methods (i.e. IHC and CBA), increasing the strength of the result. Moreover, a portion of patients with NMO or NMO spectrum diseases are negative for anti-AQP4 antibodies [3], [27], [39]. In these patients, other autoimmune conditions such as paraneoplastic neurological disorders or CTD may be present [10], [40]. Most of the antibodies associated with these conditions can be detected by IHC on monkey tissue sections, but not in the CBA. Therefore, a combination of CBA and IHC is highly recommendable as it permits to make use of the advantages of both methods.

Based on the present cohort, the assay yielded a very high sensitivity (95%) for NMO. Jarius et al. previously evaluated part of this kit (AQP4-transfected HEK cells) and found a slightly lower sensitivity (78%) for NMO in their cohort [41]. In a smaller study from our laboratory, we had found a 100% sensitivity for NMO [36]. Kim et al. and Waters et al. recently reported a sensitivity of 78% and 60%, respectively, using the same CBA [42], [43]. Importantly, however, all five studies consistently found a specificity of 100% (based on a total of 357 controls).

Differences in samples size could be responsible for the differences in sensitivity rates found between the various studies, and the lower size of our study compared to previous ones could thus represent a potential methodological limitation of our work. However, the differences obtained between larger studies (78% in two independent studies by Jarius et al. [N = 32] [41] and Kim et al. [N = 65] [42], versus 60% in the study by Waters et al. [N = 35] [43]) suggests that factors other than samples size (age? sex? genetic background? case ascertainment?) may possibly play a role as well. Larger multicentric studies with homogeneous inclusion and selection criteria are needed to definitely assess the frequency of anti-AQP4 antibodies in NMO.

4/61 patients (1 = NMO, 3 = MS) analyzed in this study were younger than 18 years at the time of blood sampling. However, previous studies [44], [45] have found a frequency of NMO-IgG/anti-AQP4 in pediatric cohorts similar to that in adult patients. Therefore, we consider it unlikely that the inclusion of 4 pediatric patients in the present study has relevantly influenced our results.

It could have been a potential limitation that the clinical and radiological criteria were evaluated by the treating physicians and obtained by us by means of a questionnaire. However, the high sensitivity and 100% specificity found in this study, which was performed in a blinded fashion, strongly argues against uncertainties in patient classification in the present study.

After submission of our study, a very interesting comparison of seven NMO-IgG/AQP4-IgG assays, including the CBA evaluated here, was published [43]. The authors found that the CBA and an in-house flow cytometry assay based assay (University of Oxford) “were the most sensitive assays”, but pointed out that the expertise and resources required to perform the flow cytometry assays would “preclude its use in small-scale clinical diagnostic laboratories”. In this study, the CBA was also compared to a commercially available ELISA (RSR, UK). The authors found that the ELISA had a slightly lower sensitivity when compared to the Euroimmun CBA (CBA-E), which could be improved by using a cut-off value lower than the one recommended by the manufacturer; however, they concluded that sera yielding values below the manufacturer's cut-off “*would require confirmatory specificity testing by CBA-E*”.

High intra- and inter-laboratory assay reproducibility is an important pre-requisite for clinical trials. Therefore, we tested 91 serum samples in our laboratory (by two different operators) and, in addition, 9 samples in parallel in our laboratory and, in a blinded fashion, at the Institute for Experimental Immunology, affiliated to Euroimmun, in Luebeck, Germany. We found a 100% concordance between results (K = 1).

In conclusion, the assay evaluated in the present study is potentially suitable for future multi-centre studies in NMO because of its very high sensitivity, specificity, and reproducibility. We therefore strongly recommend including this assay in upcoming trials comparing the diagnostic performance of the various methods currently available for the detection of anti-AQP4 antibodies.
